# *DgCspC* gene overexpression improves cotton yield and tolerance to drought and salt stress comparison with wild-type plants

**DOI:** 10.3389/fpls.2022.985900

**Published:** 2022-09-06

**Authors:** Wenwen Xia, Jiahang Zong, Kai Zheng, Yuan Wang, Dongling Zhang, Sandui Guo, Guoqing Sun

**Affiliations:** ^1^Biotechnology Research Institute, Chinese Academy of Agricultural Sciences, Beijing, China; ^2^Hainan Yazhou Bay Seed Lab, Sanya, China; ^3^College of Agriculture, Xinjiang Agricultural University, Urumqi, China

**Keywords:** *Deinococcus gobiensis*, cold shock proteins C (*DgCspC*), cotton, abiotic stress resistance, molecular breeding

## Abstract

Drought and high salinity are key limiting factors for cotton quality and yield. Therefore, research is increasingly focused on mining effective genes to improve the stress resistance of cotton. Few studies have demonstrated that bacterial *Cold shock proteins* (*Csps*) overexpression can enhance plants stress tolerance. Here, we first identified and cloned a gene *DgCspC* encoding 88 amino acids (aa) with an open reading frame (ORF) of 264 base pairs (bp) from a *Deinococcus gobiensis I-0* with high resistance to strong radiation, drought, and high temperature. In this study, heterologous expression of *DgCspC* promoted cotton growth, as exhibited by larger leaf size and higher plant height than the wild-type plants. Moreover, transgenic cotton lines showed higher tolerance to drought and salts stresses than wild-type plants, as revealed by susceptibility phenotype and physiological indexes. Furthermore, the enhanced stresses tolerance was attributed to high capacity of cellular osmotic regulation and ROS scavenging resulted from *DgCspC* expression modulating relative genes upregulated to cause proline and betaine accumulation. Meanwhile, photosynthetic efficiency and yield were significantly higher in the transgenic cotton than in the wild-type control under field conditions. This study provides a newly effective gene resource to cultivate new cotton varieties with high stresses resistance and yield.

## Introduction

Drought and salt significantly threaten plant growth, thus causing serious damage to crops and limiting yield and quality (Basu et al., 2016). Salt damage affects about 20% of the world’s cultivated land and nearly half of the irrigated land (Zhu, 2001). Cotton is an important economic crop and is mainly grown in arid and semi-arid areas in China (Fradin and Thomma, 2006; Shaban et al., 2018). Xinjiang, the main cotton-producing region in China, has a saline-alkali land area of 110,000 km^2^, accounting for about one-third of the country’s saline-alkali land area. Therefore, effective drought- or salinity-resistant genes should be identified and incorporated in genetic engineering programs to generate drought-or salinity-resistant cotton genotypes.

Desert microbes are exposed to intense solar radiation, cycles of extreme temperatures and drought. The extreme environment can cause DNA and protein damage, which is fatal to most organisms. Desert bacteria evolve with time to protect their DNA and proteins from damage or effectively repair them (Heinemann and Roske, 2021). The Institute of Biotechnology, Chinese Academy of Agricultural Sciences published the genome of *D. gobiensis I-0* in 2012. The genome of *D. gobiensis I-0* contains seven replicons: a 3.1 Mb main chromosome and six plasmids from 433 to 53 kb. GenBank accession numbers for the main chromosome and plasmids P1–P6 are from CP002191 to CP002197 (Yuan et al., 2012). About half of the genes in the genome encode proteins with unknown functions. However, *D. gobiensis I-0* plays a crucial role in resisting extreme environment of the Gobi Desert and may provide genetic resources for stress resistance. A previous study cloned *CspC* from *D. gobiensis I-0*. Csps are widely found in various organisms, including animals, plants, and microorganisms, except archaea (Weinberg et al., 2004).

Cold shock proteins (Csps) are RNA molecular chaperones in bacteria, containing primitive cold-shock domains that bind to nucleic acids, prevent RNase degradation of mRNA, and correct the misfolding of mRNA (Kentaro and Ryozo, 2012). Nine members of the *Csp* gene family (*CspA*-*CspI*) have been identified in *Escherichia coli* (Yamanaka et al., 1994). The *Csp* gene of *E. coli* and *Bacillus subtilis* can significantly improve the resistance of transgenic plants to abiotic stresses, such as drought (Castiglioni et al., 2008; Kim et al., 2009; Pingli et al., 2013; Choi et al., 2015; Yu et al., 2017). The Csps involved in various physiological stress responses of cells, including antioxidant protection, DNA damage repair and pigment synthesis through direct or indirect regulation (Faßhauer et al., 2021). Genomic sequence analysis has revealed that *D. gobiensis I-0* contains an only Csp homologous protein-coding gene (*CspC*) that is 69% similar to the amino acid sequence of *B. subtilis CspB* (Yuan et al., 2009).

Sequence structure analysis revealed that the amino acid sequence of *CspC* from *D. gobiensis I-0* is similar to that of Csp from other bacteria, all of which consist of five reverse-parallel β chains. The expression of *CspC* and *CspE* in *E. coli* is induced by temperature changes. However, *CspD* is expressed only under nutrient deficiency or steady-state stress (Yamanaka and Inouye, 1997; Yamanaka et al., 1998). Csp of *Listeria monocytogenes* participates in stress response after salt shock (Schmid et al., 2009). *CspA* of *Brucella* is involved in response to stress and virulent infestation (De La Garza-García et al., 2021). *Enterococcus* contains four cold-excited proteins, of which *CspR* is involved in bacterial virulence infestation and bacterial growth (Michaux et al., 2012). Csps in most bacteria are present in multiple copies and are induced in different ways. In contrast, *D. gobiensis I-0* contains only one Csp., implying that *DgCspC* may be induced by many different conditions, and potentially regulates multiple pathways. Previous study showed that the *CspC* expression can significantly improve the growth of *E. coli* cells under nutrient deficiency (Mingkun, 2011). In that study, a *CspC* gene expression vector driven by CaMV 35S promoter was constructed and was transformed into tobacco by *Agrobacterium*-mediated method. Transgenic tobacco lines grew normally under drought stress, while wild-type tobacco growth was retarded, indicating that this gene potentially enhances plant drought stress resistance (Mingkun, 2011). Studies on *Csp* gene in bacteria have mainly focused on *E. coli* and model plants, such as *Arabidopsis thaliana* and tobacco. However, no study has reported on the stress-resistant function of *Csp* gene in transgenic crops.

As an important oil and fiber crop, cotton is classified as moderately drought and salt tolerant crop (Zhang et al., 2013). To overcome salt and drought stresses, maintenance of cellular ion and osmotic pressure balance is the main adaptive mechanism in cotton (Naidoo and Naidoo, 2001; Rontein et al., 2002; Quan et al., 2004; Ding et al., 2010; Dai et al., 2014). Many osmoprotectants, such as amino acids, sugars, glycine betaine, polyols, and polyamines have been identified for positive involving drought and salt tolerance. It has been observed high glycine betaine and proline in response to salinity tolerance in transgenic Choline monooxygenase (CAM) gene cotton lines or wild cotton plants (Meloni et al., 2001; Zhang et al., 2009). Furthermore, high betaine could maintain the integrity of membranes against stresses and scavenging ROS (Annunziata et al., 2019). Therefore, both *GhABF3* and *GhMPK3* overexpression increases cotton tolerance to salt and drought through improving antioxidant activities (Sadau et al., 2021; Zhang et al., 2022). As an RNA molecular chaperones, Csps could maintain these RNA and improve these proteins content to enhance cotton resistant to extensive stresses.

In this study, genetic engineering technology was used to transfer the *DgCspC* gene into cotton. Subsequently, the transgenic cotton lines were self-bred to T4 generation to obtain stable lines for subsequent experiments. The *DgCspC* expression could promote growth of cotton with larger leaf size and higher plant height through improving the photosynthesis rate. The characteristic resulted in the cotton yield being significantly increased. Moreover, the transgenic cotton lines showed higher tolerant to drought and salinity stresses. Proline is key in plant response to stress (Brauc et al., 2012). *Csp* participates in osmotic stress, pigment regulation and nutritional stress regulation. Betaine is a key osmotic regulatory substance (Pukale et al., 2021). The enhanced tolerance was attributed to the proline and betaine synthesis promoted by the *DgCspC* expression. This study aimed to explore whether heterologous expression of *DgCspC* gene can improve drought and other abiotic stress resistance in cotton.

## Materials and methods

### Generation and molecular characterization of transgenic cotton expressing *DgCspC* gene

Binary expression vector pCAMBIA2300*-DgCspC* containing *CspC* gene under the control of CaMV 35S promoter was transformed into *G. hirsutum* R15 *via Agrobacterium*-mediated method. CTAB method was used to extract DNA from the leaves of the transgenic lines. The transformants were screened *via* PCR to confirm the presence of *DgCspC* gene. Transgenic cotton lines expressing *DgCspC* gene (T1) were acclimatized and then transferred to the field. Kanamycin (mass/volume ratio: 7,000 ppm) was sprayed on the transgenic cotton plants at the seedling stage. DNA was extracted from the leaves of the T2 progenies to confirm the presence of the *DgCspC* gene. Until the stably expressed T4 transgenic *DgCspC* line was harvested. The T4 generation cotton plants were planted in the field and indoor greenhouse, and the PCR positive lines were used for follow-up experiments.

Total RNA was extracted from cotton leaves using an RNAprep Pure Plant kit (Tiangen, Beijing, China), according to the manufacturer’s instructions. The RNA was then used for cDNA synthesis using a cDNA conversion kit obtained from Transgene, Beijing, China (EasyScript First-strand cDNA Synthesis SuperMix). Real-time quantitative forward and reverse primers specific for *DgCspC* were designed using Beacon Designer software. Real-time quantitative PCR analyses were performed on the LightCycler® 96 System (Roche Diagnostics Corporation) using SYBR Premix Ex Taq II (TAKARA). The *G. hirsutum UBQ7* gene were used as internal controls. The primers used are listed in Supplementary Table S2. Relative expression level was calculated using the 2^−ΔΔCT^ method (Livak and Schmittgen, 2001).

### Cotton phenotype observation and stress treatment under laboratory conditions

Experiments were performed on the seedlings of T4 generation. Seeds of transgenic and wild-type cotton plants were simultaneously planted in pots before moving them to the greenhouse with conditions of a 16-h light/8-h dark photo period, constant temperature 30°C, 12,000 lux of light intensity and 30% of relative humidity. When the eighth leaf of cotton appears, measure the plant height and the surface area of all leaves with a ruler and a leaf area meter. We evaluated the expression level of cotton growth and development response genes *CYCA3;1* (Gh_D06G022400.1), *CYCB1;1*(Gh_D05G255500.1), *EOD*(Gh_D11G228700.1), *AN3*(Gh_D12G061800.1), *EBP1*(Gh_A09G186000.1), and *GRF5*(Gh_D13G220900.1) through RT-qPCR in the control and overexpressed plants, using the *G. hirsutum UBQ7* gene as the internal control. The primers used are listed in Supplementary Table S2.

Plump seeds of *DgCspC* transgenic T4 generation and control R15 were selected, and the nutrient soil and vermiculite were mixed evenly with tap water in the ratio of 3:1, and placed in flowerpots with the same specifications. During the growth of cotton plants, MS nutrient solution was watered every other week until 4 leaves grew. *DgCspC* transgenic cotton and control cotton lines with the same development status were selected for experimental grouping. Each group contained 1 control cotton plant and 2 transgenic cotton lines. After the nutrient soil in the pot was fully soaked with tap water, drought stress treatment was carried out. The stress experiment was carried out for a total of 25 days. During the drought stress experiment, each group of cotton leaves were taken and placed in −80°C refrigerator at the same time in each period to determine the corresponding physiological indicators.

Salt stress was initiated by watering the plants with 250 mM of sodium chloride (NaCl) for 12 days (Magwanga et al., 2018a). Cotton leaves of each group were placed in a −80°C refrigerator after treatment for physiological indicator analysis. The phenotypic changes of cotton under stress were continuously observed, recorded, and photographed.

### Measurement of physiological and biochemical indexes

All samples were collected with three biological replicates before treatment, and post stress treatment. We analyzed the physiological and morphological traits of the plants. Malondialdehyde content, relative water content and relative electrical conductivity of leaf extracts were measured as described by previous researchers (Xia et al., 2021). Betaine and proline contents in cotton leaves were determined using HPLC-MS (Shimadzu LC20AD-API 3200MD TRAP). Sample pretreatment was conducted as follows: Pure water was added to the samples, then vortexed for 10 min, followed by ultrasound for 10 min and centrifugation at 13,200 r/min for 4 min. The arginine and proline samples were precipitated with 50 ul and 150 acetonitrile, then vortexed for 2 min, centrifuged at 13,200 r/min for 4 min to obtain supernatant.

### Expression profile of genes related to betaine and proline synthesis

We evaluated the expression level of genes related to betaine and proline synthesis-responsive, such as HMT-2 (Gh_D08G068000.1), At4g29890(Gh_A05G415500.1), ALDH10A8(Gh_A11G044900.1), ALDH10A8(Gh_D11G045100.1), ALDH10A8(Gh_A07G068600.1), HMT-2(Gh_A08G073000.1), At4g29890(Gh_D04G006200.1), HMT3(Gh_A02G039800.1), HMT3(Gh_D02G045900.1), At4g29890(Gh_A08G188100.1), Gh_D11G286600(Gh_D11G286600.1), Gh_A11G286400(Gh_A11G286400.1), ODC(Gh_D11G286700.1), AIH(Gh_D07G063100.1), ALDH2B4(Gh_A07G245500.1), Gh_D07G028100(Gh_D07G028100.1),Gh_A03G228600(Gh_A03G228600.1), Gh_D02G245200(Gh_D02G245200.1), ASP3(Gh_A07G027000.1), Gh_A13G098000(Gh_A13G098000.1), POX2(Gh_A07G195 200.1), ALDH3H1(Gh_D06G049900.1), AIH(Gh_A07G062700.1), SPMS(Gh_A12G275700.2), Gh_D13G104800(Gh_D13G104800.1), ASP3(Gh_D07G028200.1), PAO5,(Gh_A05G023200.1), ARB_02965 (Gh_D01G183300.1), ALDH2B4(Gh_A12G299800.1), ALDH3F1(Gh_D05G071600.1), PAO5(Gh_D05G031600.1), ALDH2B4(Gh_D12G293500.1), P4H4(Gh_D09G248800.1), PAO1(Gh_A08G165800.1), POX2(Gh_D07G193100.1) and P4H7(Gh_D06G014100.1) through RT-qPCR in the tissues of control and overexpressed plants, using the *G. hirsutum UBQ7* gene as the internal control. The primers used are listed in Supplementary Table S2. As demonstrated by previous researchers, these genes are highly upregulated in various cotton and *Arabidopsis* plants’ tissues and enhance the synthesis of betaine and proline.

### Determination of agronomic traits and photosynthetic rate in the field

The field sowing experiment was carried out between *DgCspC* transgenic cotton and the control (43°335′ N, 84°976′ E) in Shawan County, Xinjiang based on the random distribution principle (plot area; 2.25 m * 5 m). Each plot was replicated three times. Photosynthetic parameters including net photosynthetic rate, intercellular CO_2_ concentration, stomatal conductance, transpiration rate, vapor pressure deficiency, and net water use efficiency were measured during full flowering using a portable gas exchange photosynthetic GFS-3000 (WLAZ, Germany) following the system instructions. In addition, the related agronomic traits including plant height, number-of fruit branches, bell number, and bell weight were assessed at the full bolling stage, while the yield was quantified during the harvest stage.

### Statistical analysis

SPSS 18.0 statistical software (SPSS Inc. Chicago, IL, United States) was used to analyze and process the data. Origin9.0 software was used to plot the data. Data in the charts are expressed as mean ± standard error. Asterisks (* and **) represent *p* ≤ 0.05 and *p* ≤ 0.01, respectively.

## Results

### Identification and expression analysis of transgenic cotton expressing *DgCspC* gene

**Figure 1:**
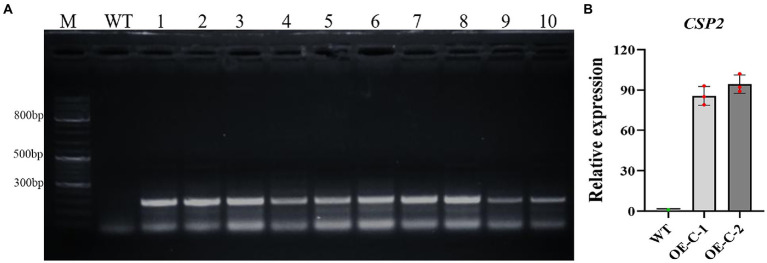
Polymerase chain reaction (PCR) and quantitative RT-PCR (qRT-PCR) analysis of *CspC* transgenic cotton. **(A)** Gel image showing PCR identification of *CspC* T4 transgenic cotton; **(B)** *CspC* gene expression profile in OE-C-1/OE-C-2 lines.

Tissue culture transgenic cotton seedlings expressing *DgCspC* gene were grafted to the stock and cultivated in a greenhouse to obtain cotton plants with *DgCspC* overexpression in the T1 generation. Cotton seeds of the T1 generation were successively self-bred in Hainan and Xinjiang provinces to obtain T4 stable lines. The presence and expression of the *DgCspC* gene in the T4 stable lines were confirmed by kanamycin selection and PCR identification (Figure 1A). Lines 1 and 2, whose samples exhibited the right band size after gel electrophoresis were selected and named OE-C-1 and OE-C-2, respectively, for subsequent analysis. The *DgCspC* gene was stable and highly expressed in OE-C-1/OE-C-2 lines based on real-time fluorescence quantitative analysis (Figure 1B). These results suggest that *DgCspC* gene was stably expressed in T4 transgenic cotton.

### *DgCspC* gene promote cotton growth

**Figure 2:**
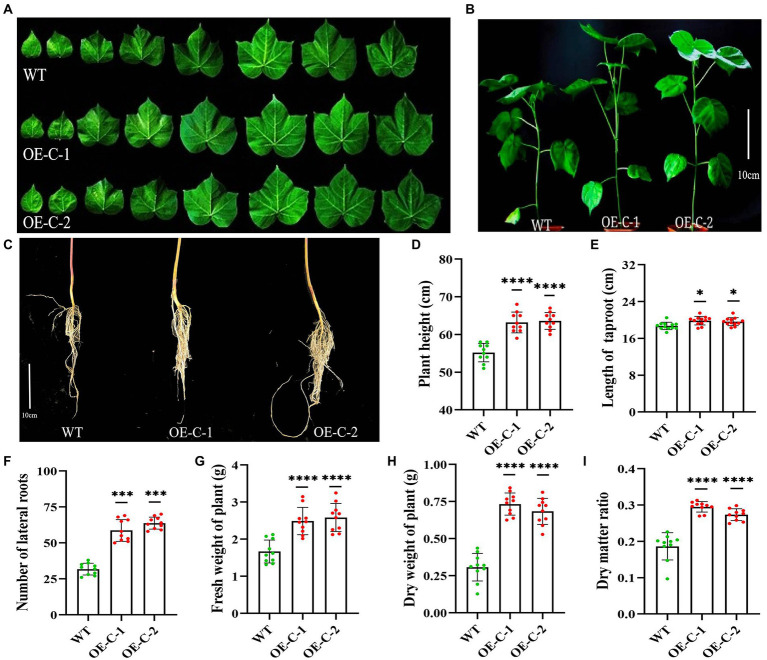
Growth analysis of *DgCspC* transgenic cotton lines. **(A)** Comparison of leaf size between transgenic and wild-type cotton lines; **(B)** growth phenotype of wild type and transgenic cotton lines; **(C)** root morphology between wild type and transgenic cotton lines; **(D)** height comparison between wild type and transgenic cotton lines; **(E)** length of taproot; **(F)** number of lateral roots; **(G)** fresh weight of plant; **(H)** dry weight of plant; **(I)** dry matter ratio. Asterisks indicate significant differences between WT and the OE-C-1/ OE-C-2 transgenic lines. (^*^p < 0.05, ^***^p < 0.001, ^****^p < 0.0001 for comparisons between the transgenic lines and wild-type plants by Student’s *t*-tests).

The harvested T4 generation OE-C-1/OE-C-2 cotton seeds and wild-type R15 seeds were simultaneously planted in the greenhouse and cultured for 30 days. The transgenic cotton plants exhibited significantly higher growth rate compared with the wild-type, while their leaves gradually increased based on the area of the 1st–8th leaves (Table 1). The area of the third leaf of OE-C-1/OE-C-2 lines was significantly larger than that of the wild type (Table 1; Figure 2A). The average plant height of OE-C-1/OE-C-2 lines was significantly higher than that of the wild type (Figures 2B,D). The taproot length of OE-C-1/OE-C-2 lines was slightly longer than that of the wild type, but the number of lateral roots was significantly higher than that of the wild type (Figures 2C,E,F). Cotton lines expressing *CspC* gene showed significantly higher nutritional growth relative to their wild-type counterparts. The fresh weight and dry weight of OE-C-1/OE-C-2 lines were significantly higher than those of the wild type (Figures 2G–I). These data suggest that *DgCspC* expression promotes the growth and leaf development of cotton.

**Table 1:** Cotton leaf area (cm^2^).

	First leaf	Second leaf	Third leaf	Fourth leaf	Fifth leaf	Sixth leaf	Seventh leaf	Eighth leaf
WT	28.2 ± 0.57	29.3 ± 0.20	35.73 ± 0.50	59.13 ± 0.40	81.37 ± 1.15	105.6 ± 0.96	121.97 ± 1.55	123.97 ± 0.42
OE-C-1	28.63 ± 0.42^ns^	29.67 ± 0.38^ns^	56.97 ± 1.31^**^	69.3 ± 0.95^***^	109.2 ± 0.70^***^	130.67 ± 0.85^***^	139.77 ± 0.45^***^	142.73 ± 0.40^***^
OE-C-2	28.33 ± 0.32^ns^	30.23 ± 0.15^*^	61.47 ± 1.15^***^	73.73 ± 1.40^***^	111.5 ± 0.82^***^	133.73 ± 0.80^***^	143.23 ± 1.03^***^	145.37 ± 0.49^***^

Asterisks indicate significant differences between WT and the OE-C-1/OE-C-2 transgenic lines (Student’s *t*-test, ^**^p < 0.01). WT, wild type; OE, overexpression; CspC, cold shock protein C.

^*^p < 0.05, ^**^p < 0.01, ^***^p < 0.001 comparisons between the transgenic lines and wild-type plants by Student’s *t*-tests.

**Figure 3:**
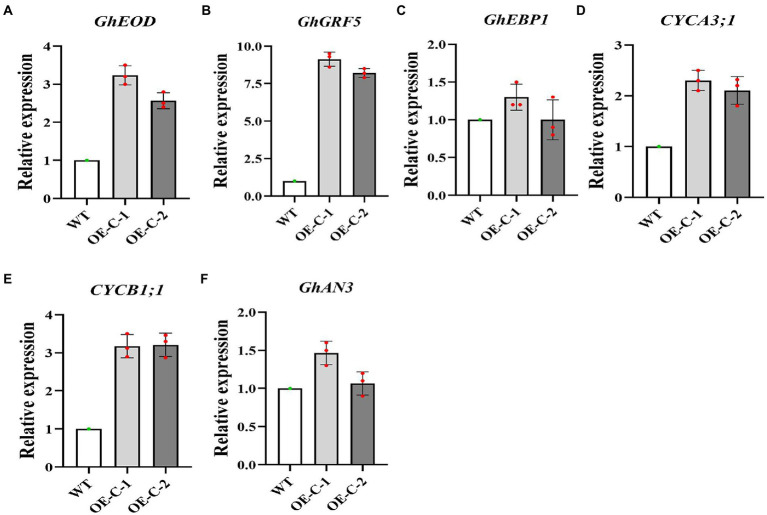
Expression analysis of genes related to growth and development in the transgenic cotton lines. **(A)** GhEDO Relative expression. **(B)** GhGRF5 Relative expression. **(C)** GhEBP1 Relative expression. **(D)** CYCA3;1 Relative expression. **(E)** CYCB1;1 Relative expression. **(F)** GhAN3 Relative expression.

In order to further study the reasons for the vigorous growth of cotton, we selected six genes related to cotton cell development for real-time quantitative expression analysis. Quantitative analysis revealed that the Type A and type B cyclins CYCA3;1 and CYCB1;1 were upregulated in the transgenic lines and regulated cell growth (Figures 3D,E). Growth and development regulatory genes, including *EOD*, *AN3*, *EBP1*, and *GRF5* were further selected for quantitative analysis. The results showed that *EOD* and *GRF5* genes were significantly upregulated in the transgenic cotton lines, suggesting that they facilitated the rapid growth of the transgenic cotton lines (Figures 3A–C,F). These data suggest that the rapid growth and leaf cell development of cotton were closely related to the upregulated expression of *CYCA3;1*, *CYCB1;1*, *EOD*, *AN3*, *EBP1*, and *GRF5*.

### *DgCspC* gene improves cotton tolerance to drought and salt stress

**Figure 4:**
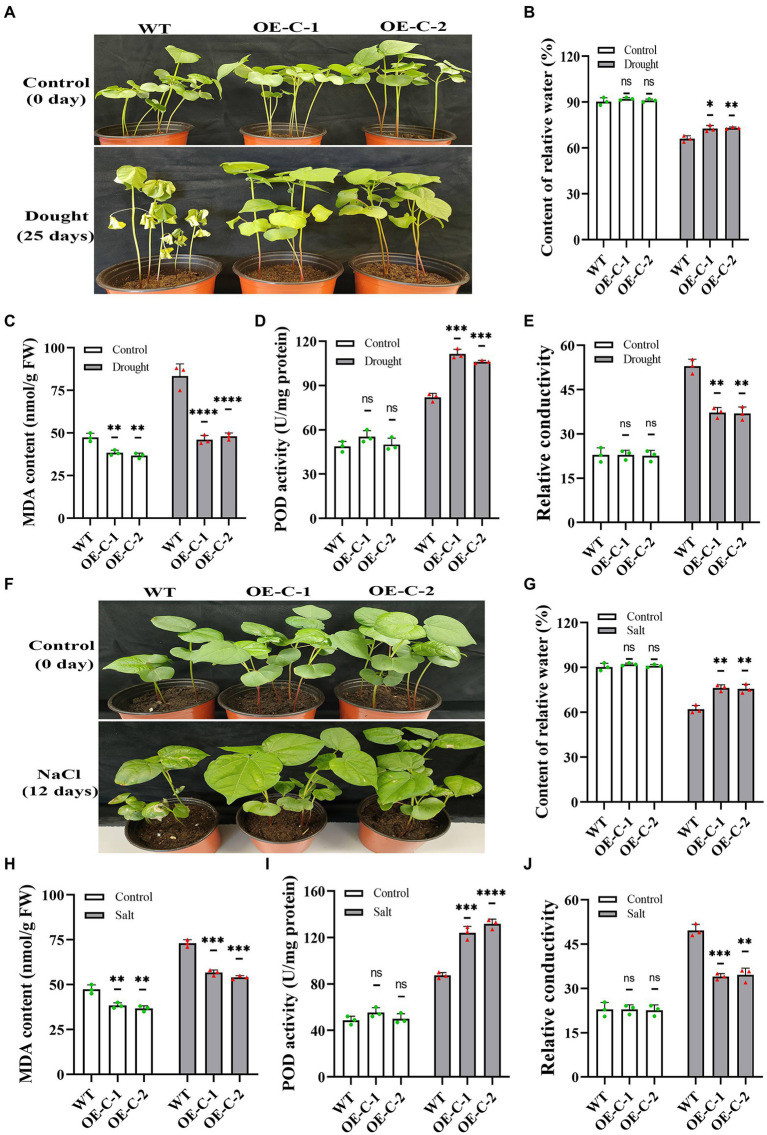
Phenotype and physiological indexes of *DgCspC* transgenic cotton under drought and salt stress. **(A)** Phenotype of transgenic and wild-type cotton line under natural drought and rehydration; **(B)** relative water content; **(C)** MDA content; **(D)** POD content; **(E)** relative conductivity; **(F)** phenotype of transgenic and wild-type cotton lines under salt treatment; **(G)** relative water content under salt stress. **(H)** MDA content under salt stress; **(I)** POD content under salt stress; **(J)** relative conductivity under salt stress; Asterisks indicate significant differences between WT and the OE-C-1/ OE-C-2 transgenic lines. (^*^p < 0.05, ^**^p < 0.01, ^***^p < 0.001, ^****^p < 0.0001 for comparisons between the transgenic lines and wild-type plants by Student’s *t*-tests).

The transgenic cotton seedlings that grew naturally for 15 days were treated with drought to further identify the stress resistance function of *DgCspC* gene. The wild-type cotton leaves showed serious wilting and shriveled together when the natural drought lasted for 25 days (Figure 4A). However, the leaves of OE-C-1/OE-C-2 cotton lines were slightly dehydrated and grew normally. For instance, the cotton lines OE-C-1/OE-C-2 grew well and had a larger leaf size and higher plant height than the control group. *DgCspC* transgenic cotton showed a strong drought resistance phenotype. Similar results were obtained at the cellular physiological level in *DgCspC* transgenic cotton. The relative water content, MDA content, POD activity, and relative conductivity were measured using cotton leaves under normal growth versus those under drought treatment for 25 days. Relative water content and POD activity were significantly higher in OE-C-1/OE-C-2 lines than in the wild type after drought stress (Figures 4B–E). In contrast, the MDA content and relative conductivity were significantly lower in OE-C-1/OE-C-2 lines than in the wild type, indicating that cell damage was significantly lower in *DgCspC* transgenic cotton than in R15 wild type after drought stress.

The leaves of wild-type cotton turned yellow and withered after treatment with 250 mM NaCl for 12 days. However, the leaves of the transgenic cotton were slightly yellow after the treatment (Figure 4F). The damage to the membrane system was significantly lower in transgenic cotton than in the wild-type cotton under salt stress based on physiological indexes. The relative water content of cotton leaves decreased significantly after salt treatment, but the relative water content of OE-C-1/OE-C-2 lines was still significantly higher than that of wild type (Figure 4G). The MDA content of *DgCspC* overexpression lines was significantly higher than that of wild type before and after salt treatment (Figure 4H). After 200 mM NaCl treatment, the POD activity of the OE-C-1/OE-C-2 lines was significantly higher than that of the wild type, and the relative conductivity was significantly lower than that of the wild type (Figures 4I,J). Overall, these results indicate that overexpression of *DgCspC* gene can improve drought and salt stress tolerance in cotton.

### Analysis of proline and betaine contents and gene expression level

**Figure 5:**
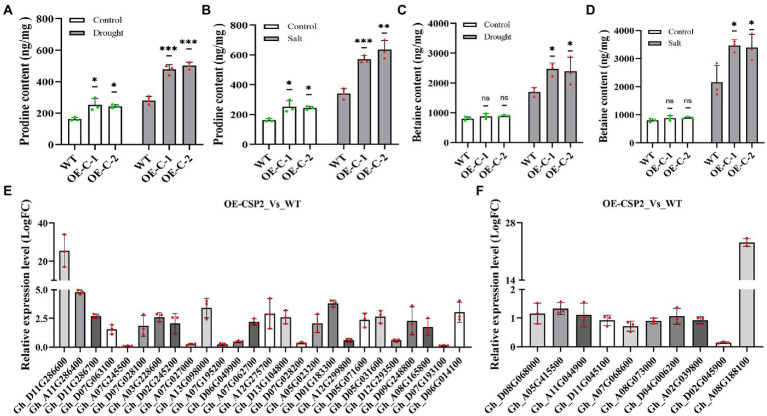
Analysis of proline and betaine contents and expression of related genes in transgenic cotton overexpressing *DgCspC* gene under drought or salt stress. **(A)** Proline content under drought stress; **(B)** proline content under salt stress; **(C)** betaine content under drought stress; **(D)** betaine content under salt stress; **(E)** expression of proline synthesis-related genes; **(F)** expression of betaine synthesis-related genes. (^*^p < 0.05, ^**^p < 0.01, ^***^p < 0.001 for comparisons between the transgenic lines and wild-type plants by Student’s *t*-tests.)

To understand the reason that *DgCspC* expression enhance cotton tolerance to drought and salinity stresses, the proline and betaine contents of both transgenic cotton lines and WT lines were measured under the normal and stresses conditions. In this study, the proline content was significantly higher in OE-C-1/OE-C-2 lines than in wild type after drought and salt stress treatments (Figures 5A,B). Herein, betaine content in cotton leaves was determined using high-performance liquid chromatography mass spectrometry. The results showed that betaine content was significantly higher in OE-C-1/OE-C-2 cotton lines than in the control group after drought and salt treatments. Under normal conditions, the betaine content of transgenic lines was slightly higher than that of wild-type lines, but not significantly (Figures 5C,D). Increased betaine content in cells can enhance osmotic regulation and drought and salt tolerance of plants.

Quantitative analysis revealed that several genes related to proline and betaine synthesis were upregulated in *DgCspC* transgenic cotton. Specifically, 26 proline synthesis-related genes were screened from the transgenic cotton lines, of which 18 genes were significantly upregulated (Figure 5E). Similarly, proline content was higher in the transgenic cotton leaves than in the wild type. Secondly, 10 betaine synthesis-related genes were screened from *DgCspC* overexpression cotton of which eight genes were upregulated. The upregulation range was slightly small (Figure 5F). Betaine content was not significantly different between transgenic cotton and wild type under normal conditions. However, betaine content was significantly higher in transgenic cotton than in wild type after drought and salt stress treatments (Figures 5C,D). These data showed that the upregulated expression of most genes in the proline and betaine synthesis pathway led to the increase in proline and betaine contents in transgenic cotton leaves.

### *DgCspC* transgenic cotton exhibits strong photosynthetic rate under field conditions

**Figure 6:**
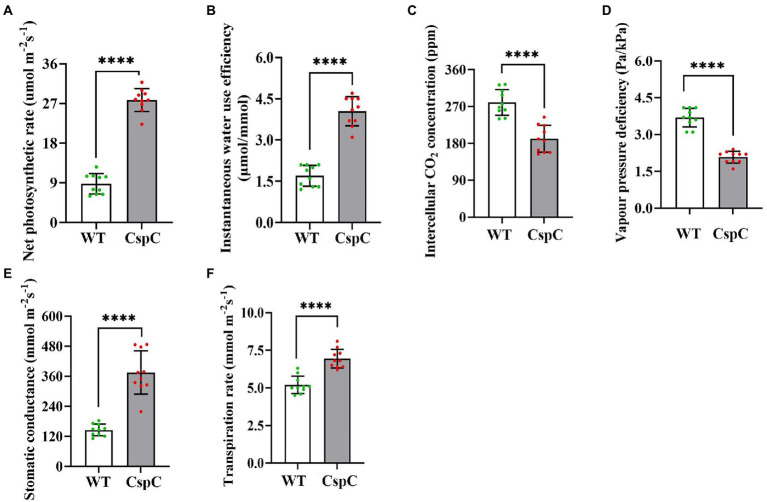
Photosynthetic efficiency of *DgCspC* overexpression cotton. **(A)** Net photosynthetic rate; **(B)** instantaneous water use efficiency; **(C)** intercellular CO_2_ concentration; **(D)** vapor pressure deficiency; **(E)** stomatal conductance; **(F)** transpiration rate. Asterisks indicate significant differences between WT and the OE-C-1/ OE-C-2 transgenic lines. (^****^p < 0.0001 for comparisons between the transgenic lines and wild-type plants by Student’s *t*-tests).

In order to further observe the characters and yield of transgenic cotton in the field, the field sowing experiment was carried out between *DgCspC* transgenic cotton and the control. Photosynthesis represents the biomass and yield of the plant. For the photosynthetic assay, penultimate leaves from transgenic cotton plants were obtained from 10 plants per line (*DgCspC*-transgenic lines and control recipient plants). The photosynthetic parameters, including net photosynthetic rate, intercellular CO_2_ concentration, stomatal conductance, transpiration rate, and net water use efficiency were significantly higher in the *DgCspC*-transformed cotton than in control when grown normally in the field (Figure 6) except for vapor pressure deficiency (VPD). As a result, the transgenic cotton plants had better growth conditions and agronomic trait performance than the control.

The related agronomic traits were investigated at the full bolling stage, while yield was investigated at the harvest stage. Transgenic cotton lines exhibited higher plant height, more fruit branches, more bolls, and higher yield (which increased by 9.8%, 37%, and 30.08%, respectively) under field conditions than the wild type (Table 2). These data showed that the overexpression of *DgCspC* gene significantly increased cotton yield.

**Table 2:** Agronomic traits and yield of cotton in the field.

Strain	Plant height (cm)	Number-of fruit branches (/plant)	Bell number (/plant)	Bell weight (g)	Seed-cotton weight (g)
WT	66.75 ± 4.96	6.75 ± 1.09	4.92 ± 0.86	78.12 ± 18.84	90.79 ± 4.65
OE-CspC	73.33 ± 2.96^***^	9.25 ± 1.01^****^	6.4 ± 1.34^**^	102.27 ± 21.45^**^	113.82 ± 8.14^****^

Asterisks indicate significant differences between WT and the OE-C-1/OE-C-2 transgenic lines (Student’s *t*-test, ^**^p < 0.01). WT, wild type; OE, overexpression; CspC, Cold shock protein C.

^**^p < 0.01, ^***^p < 0.001, ^****^p < 0.0001 for comparisons between the transgenic lines and wild-type plants by Student’s *t*-tests.

## Discussion

*Csps* are found in various bacteria. All *Csps* share common features that play a critical role in the survival of bacteria in extreme environments (Amir et al., 2018). Efficient use of carbon sources in a competitive environment is essential for bacterial survival. As a result, bacteria have evolved a complex control system for efficient energy use (Chen, 2013). *Csp* genes act as molecular chaperones that maintain mRNA stability under abiotic stresses, such as drought and low temperature (Keto-Timonen et al., 2016). *Csp* genes can regulate transcription and translation efficiency by binding to DNA or RNA, improve freezing resistance of biofilms by encoding new proteins, and participate in signal transduction (Ermolenko and Makhatadze, 2002). Our study revealed that *CYCA3;1*, *CYCB1;1*, *EOD* and *GRF5* were significantly upregulated in the *DgCspC* transgenic lines and regulated cell growth. Cell cycle plays an important role in the growth and development of multicellular organisms (Dewitte and Murray, 2003). Type A and type B cyclins (CYCAs/CYCBs) are mitotic cyclins. Studies have shown that overexpression of *CYCA3* in tobacco can induce cell division in shoot tip meristem and leaf primordium (Wyrzykowska et al., 2002). Growth-Regulating Factors (*GRFs*) belong to a small plant specific transcription factor (TF) family. *GRFs* function in regulating leaf (Kim et al., 2003) and stem development, shoot apical meristem development (Kim and Lee, 2006), leaf primordia formation (Horiguchi et al., 2005), and leaf size and longevity (Debernardi et al., 2014). Growth-Regulating Factors (*GRFs*) plays an important role in regulating leaf and stem development, shoot tip meristem development, leaf primordium formation, and leaf size (Kim et al., 2003; Horiguchi et al., 2005; Kim and Lee, 2006; Debernardi et al., 2014). Overexpression of *GRFs* promotes the expression of cell cycle genes and auxin response genes, resulting in increased cell numbers and expansion of leaves (Omidbakhshfard et al., 2015; Piya et al., 2020). The *CspC* expression could cause these genes upregulated to increase expansion of cotton leaves. It was inferred that upregulated of these genes might be resulted from *DgCspC* function in transcription and translation efficiency or activating a signal pathway to induce these gene expression.

Csps in most bacteria are present in multiple copies and are induced in different ways. In contrast, *D. gobiensis I-0* contains only one *Csp.,* implying that it may be induced by many different conditions, and potentially regulates multiple pathways. A study found that cold-excited proteins are involved in the survival of cells during the stable phase (Michaux et al., 2012). Csp participates in osmotic stress, pigment regulation, nutritional stress regulation, and other cellular processes, effectively enhancing resistance to cold and osmotic stresses.

Drought and salt significantly affect the normal growth and development of plants, causing a huge loss of cotton yield (Cai et al., 2021). Plant phenotype is the most visual response to drought and salt stresses. The relative leaf water content measure water status in the plant, and reflects the metabolic activity of the tissues (Nayyar et al., 2006). In this study, transgenic cotton lines overexpressing *DgCspC* gene exhibited higher water content after stress, indicating that *DgCspC* gene can enhance the water retention of leaves under stress and improve the drought and salt resistance of the plants. Stresses, such as drought and salt can induce oxidative stress, leading to membrane peroxidation, thus damaging cells. MDA and the relative conductivity are effective indexes used to measure the degree of lipid peroxidation. The MDA content and the relative conductivity increase in plants under drought and salt stresses. In this study, the MDA content and relative conductivity were lower in the transgenic cotton than in the control plants under stress, indicating that overexpression of the *DgCspC* gene can reduce oxidative damage, consistent with the findings reported previously (Yu et al., 2017).

Excessive reactive oxygen species (ROS) in plants can cause membrane lipid peroxidation, resulting in oxidative damage to the membrane system (Zhang et al., 2005; Basu et al., 2017). Plants have various mechanisms of adapting to abiotic stress, such as accumulating some soluble sugars, amino acids, organic acids, proline, polyols, betaine, and other soluble substances (Giri, 2011; Kumar et al., 2017). Betaine resists adverse stress through osmotic regulation, scavenging ROS, maintaining biofilm stability, protecting photosynthetic mechanism, and maintaining the structure and function of macromolecular protein complexes and some enzymes (Chen and Murata, 2011). Betaine and proline are key osmoregulatory substances in plants. They enhance plant adaptation to water stress caused by drought, salinity, and other stresses. High proline content can maintain the osmotic balance of cytosol and reduce the damage to cells (Yang and Gao, 2007; Chen and Murata, 2008; Ahmad et al., 2010; Zhang et al., 2014). In this study, overexpression of *DgCspC* gene significantly increased betaine and proline contents in cotton leaves, thus enhancing the cell osmotic regulation function under drought and salt stresses. Betaine can also improve salt tolerance by strengthening CO_2_ assimilation ability of plant cells. A study showed that betaine significantly improves the photosynthetic capacity, stomatal conductance, transpiration rate, and the activity of related antioxidant enzymes in wheat plants under salt stress (Ashraf et al., 2008). Herein, the net photosynthetic efficiency was significantly higher in transgenic cotton than in wild-type, possibly due to increased betaine content under salt and drought stresses. Increased photosynthetic efficiency increased cotton yield. Therefore, *DgCspC* expression could promote transcription and translation of genes of Betaine and proline metabolisms under adverse conditions, and contributed accumulated of Betaine and proline in cotton. It was consistent with the *DgCspC* expression induced by many ways and regulating various pathways. A previous study showed that *CspC* gene of *D. gobiensis I-0* can significantly improve the resistance of *E. coli* to low temperature, high salt, drought, and other adversities (Chen, 2013). In this study, heterologous expression of *CspC* gene from *D. gobiensis I-0* in cotton accelerated the growth, yield, and resistance to drought and salt stresses. This is the first study to demonstrate the functional application of *DgCspC* gene in genetic engineering, thus is of theoretical and practical significance. Additionally, the genome of *D. gobiensis I-0* contains several functional genes and species genes. Therefore, this genome harbors important genetic resources, especially stress-related genes and damage repair genes. However, further studies should explore these genes and their practical application by clarifying their mechanism of action. Moreover, the stress-resistant genes can be used to generate new varieties of transgenic crops with stress resistance. Recent studies have shown that Csps can significantly enhance the salt tolerance of *E. coli* and *Brassica rapa* and globally regulate the expression of genes, including stress-reactive proteins and growth-related proteins (Pan et al., 2009). Therefore, Csps may provide candidate resources for developing superior transgenic plant varieties.

## Conclusion

These research findings have shown that *DgCspC* gene plays a role in enhancing drought and salt tolerance of cotton. In this study, heterologous expression of *DgCspC* promoted cotton growth, as exhibited by larger leaf size and plant height, compared with the wild-type plants. Furthermore, Proline and betaine content assays confirmed that the enhanced stress tolerance of *DgCspC* transgenic cotton was related to its osmotic regulation. In addition, comparative transcriptome analysis showed that the expression of genes related to the synthesis of betaine and proline was upregulated. Also, photosynthetic efficiency and yield were significantly higher in the transgenic cotton overexpressing *DgCspC* than in the wild-type control under field conditions. This is the first report that microbial *Csp* gene improves the stress resistance of cotton. This study provides insights into the molecular breeding of new cotton varieties with high stress resistance.

## Data availability statement

The original contributions presented in the study are included in the article/Supplementary material, further inquiries can be directed to the corresponding author.

## Author contributions

WX and SuG conceived and designed the study. JZ and KZ performed the experiments. YW and DZ prepared the figures and analyzed the data. WX wrote the manuscript. SaG and SuG performed a critical review for intellectual content. All authors contributed to the article and approved the submitted version.

## Funding

This research was funded by Hainan Yazhou Bay Seed Lab (grant nos. B21HJ8201 and B21Y10213).

## Conflict of interest

The authors declare that the research was conducted in the absence of any commercial or financial relationships that could be construed as a potential conflict of interest.

## Publisher’s note

All claims expressed in this article are solely those of the authors and do not necessarily represent those of their affiliated organizations, or those of the publisher, the editors and the reviewers. Any product that may be evaluated in this article, or claim that may be made by its manufacturer, is not guaranteed or endorsed by the publisher.

## References

[ref1] Ahmad R. Kim Y. H. Kim M. D. Kwon S. Y. Cho K. Lee H. S.. (2010). Simultaneous expression of choline oxidase, superoxide dismutase and ascorbate peroxidase in potato plant chloroplasts provides synergistically enhanced protection against various abiotic stresses. Physiol. Plant. 138, 520–533. doi: 10.1111/j.1399-3054.2010.01348.x, PMID: 20059737

[ref2] Amir M. Kumar V. Dohare R. Islam A. Ahmad F. Hassan M. I. (2018). Sequence, structure and evolutionary analysis of cold shock domain proteins, a member of OB fold family. J. Evol. Biol. 31, 1903–1917. doi: 10.1111/jeb.13382, PMID: 30267552

[ref3] Annunziata M. G. Ciarmiello L. F. Woodrow P. Dell’Aversana E. Carillo P. (2019). Spatial and temporal profile of glycine Betaine accumulation in plants Under abiotic stresses. Front. Plant Sci. 10:230. doi: 10.3389/fpls.2019.00230, PMID: 30899269 PMC6416205

[ref4] Ashraf M. Nawaz K. Athar H. R. Raza S. H. (2008). Growth enhance-ment in two potential cereal crops, Maize and Wheat, by Exogenous Application of Glycinebetaine. Switzerland: Birkhäuser Basel.

[ref5] Basu S. Giri R. K. Benazir I. Kumar S. Rajwanshi R. Dwivedi S. K.. (2017). Comprehensive physiological analyses and reactive oxygen species profiling in drought tolerant rice genotypes under salinity stress. Physiol. Mol. Biol. Plants 23, 837–850. doi: 10.1007/s12298-017-0477-0, PMID: 29158633 PMC5671459

[ref6] Basu S. Ramegowda V. Kumar A. Pereira A. (2016). Plant adaptation to drought stress. F1000Res 5:1554. doi: 10.12688/f1000research.7678.1, PMID: 27441087 PMC4937719

[ref7] Brauc S. De Vooght E. Claeys M. Geuns J. M. C. Höfte M. Angenon G. (2012). Overexpression of arginase in *Arabidopsis thaliana* influences defence responses against Botrytis cinerea. Plant Biol. 14, 39–45. doi: 10.1111/j.1438-8677.2011.00520.x, PMID: 22188168

[ref8] Cai X. Jiang Z. Tang L. Zhang S. Li X. Wang H.. (2021). Genome-wide characterization of carotenoid oxygenase gene family in three cotton species and functional identification of GaNCED3 in drought and salt stress. J. Appl. Genet. 62, 527–543. doi: 10.1007/s13353-021-00634-3, PMID: 34109531

[ref9] Castiglioni P. Warner D. Bensen R. J. (2008). Bacterial RNA chaperones confer abiotic stress tolerance in plants and improved grain yield in maize under water limited conditions. Plant Physiol. 147, 446–455. doi: 10.1104/pp.108.118828, PMID: 18524876 PMC2409020

[ref10] Chen J. (2013). Molecular mechanism of the *Escherichia coli* maltose transporter. Curr. Opin. Struct. Biol. 23, 492–498. doi: 10.1016/j.sbi.2013.03.011, PMID: 23628288 PMC3743091

[ref11] Chen T. H. H. Murata N. (2008). Glycinebetaine: an effective protectant against abiotic stress in plants. Trends Plant Sci. 13, 499–505. doi: 10.1016/j.tplants.2008.06.007, PMID: 18703379

[ref12] Chen T. H. H. Murata N. (2011). Glycinebetaine protects plants against abiotic stress: mechanisms and biotechnological applications. Plant Cell Environ. 34, 1–20. doi: 10.1111/j.1365-3040.2010.02232, PMID: 20946588

[ref13] Choi M. J. Park Y. R. Park S. J. Heung K. (2015). Stress-responsive expression patterns and functional characterization of cold shock domain proteins in cabbage (Brassica rapa) under abiotic stress conditions. Plant Physiol. Biochem. 96, 132–140. doi: 10.1016/j.plaphy.2015.07.027, PMID: 26263516

[ref14] Dai J. Duan L. Dong H. (2014). Improved nutrient uptake enhances cotton growth and salinity tolerance in saline media. J. Plant Nutr. 37, 1269–1286. doi: 10.1080/01904167.2014.881869

[ref15] De La Garza-García J. A. Bettache S. O. Lyonnais S. Eusebio E. O. Freddi L. Dahouk S. A. I.. (2021). Comparative genome-wide Transcriptome analysis of Brucella suis and Brucella microti Under acid stress at pH 4.5: cold shock protein CspA and Dps are associated With acid resistance of B microti. Front. Microbiol. 12:794535. doi: 10.3389/fmicb.2021.794535, PMID: 34966374 PMC8710502

[ref16] Debernardi J. M. Mecchia M. A. Vercruyssen L. Smaczniak C. Kaufmann K. Inze D.. (2014). Post-transcriptional control of GRF transcription factors by microRNA miR396 and GIF co-activator affects leaf size and longevity. Plant J. 79, 413–426. doi: 10.1111/tpj.12567, PMID: 24888433

[ref17] Dewitte W. Murray J. A. H. (2003). The plant cell cycle. Annu. Rev.Plant Biol. 54, 235–264. doi: 10.1146/annurev.arplant.54.031902.134836 14502991

[ref18] Ding M. Peichen H. Xin S. Meijuan W. Shurong D. Jian S.. (2010). Salt-induced expression of genes related to Na+/K+ and ROS homeostasis in leaves of salt-resistant and salt-sensitive poplar species. Plant Mol. Biol. 73, 251–269. doi: 10.1007/s11103-010-9612-9 20157764

[ref19] Ermolenko D. N. Makhatadze G. I. (2002). Bacterial cold-shock proteins. Cell. Mol. Life Sci. 59, 1902–1913. doi: 10.1007/pl00012513 12530521 PMC11337450

[ref20] Faßhauer P. Busche T. Kalinowski J. Mäder U. Poehlein A. Daniel R.. (2021). Functional redundancy and specialization of the conserved cold shock proteins in *Bacillus subtilis*. Microorganisms 9:1434. doi: 10.3390/microorganisms9071434, PMID: 34361870 PMC8307031

[ref21] Fradin E. F. Thomma B. P. H. J. (2006). Physiology and molecular aspects of Verticillium wilt diseases caused by V. dahliae and V. albo-atrum. Mol. Plant Pathol. 7, 71–86. doi: 10.1111/j.1364-3703.2006.00323.x, PMID: 20507429

[ref22] Giri J. (2011). Glycinebetaine and abiotic stress tolerance in plants. Plant Signal. Behav. 6, 1746–1751. doi: 10.4161/psb.6.11.17801, PMID: 22057338 PMC3329348

[ref24] Heinemann U. Roske Y. (2021). Cold-shock domains—abundance, structure, properties, and nucleic-acid binding. Cancers 13:190. doi: 10.3390/cancers13020190, PMID: 33430354 PMC7825780

[ref25] Horiguchi G. Kim G. T. Tsukaya H. (2005). The transcription factor AtGRF5 and the transcription coactivator AN3 regulate cell proliferation in leaf primordia of *Arabidopsis thaliana*. Plant J. 43, 68–78. doi: 10.1111/j.1365-313X.2005.02429.x, PMID: 15960617

[ref26] Kentaro E. Ryozo E. (2012). Pleiotropic roles of cold shock domain proteins in plants. Front. Plant Sci. 2:116. doi: 10.3389/fpls.2011.00116, PMID: 22639630 PMC3355641

[ref27] Keto-Timonen R. Hietala N. Palonen E. Hakakorpi A. Lindström M. Korkeala H. (2016). Cold shock proteins: A Minireview with special emphasis on Csp-family of Enteropathogenic Yersinia. Front. Microbiol. 7:1151. doi: 10.3389/fmicb.2016.01151, PMID: 27499753 PMC4956666

[ref28] Kim J. H. Choi D. Kende H. (2003). The AtGRF family of putative transcription factors is involved in leaf and cotyledon growth in Arabidopsis. Plant J. 36, 94–104. doi: 10.1046/j.1365-313X.2003.01862.x, PMID: 12974814

[ref29] Kim J. H. Lee B. H. (2006). Growth-Regulating Factor4 of *Arabidopsis thaliana* is required for development of leaves, cotyledons, and shoot apical meristem. J. Plant Biol. 49, 463–468. doi: 10.1007/BF03031127

[ref30] Kim M. H. Sasaki K. Imai R. (2009). Cold shock domain protein 3 regulates freezing tolerance in *Arabidopsis thaliana*. J. Biol. Chem. 284, 23454–23460. doi: 10.1074/jbc.m109.025791, PMID: 19556243 PMC2749119

[ref31] Kumar V. Shriram V. Hoque T. S. Hasan M. M. Burritt D. J. Hossain M. A. (2017). Glycinebetaine-mediated abiotic oxidative-stress tolerance in plants: physiological and biochemical mechanisms. Switzerland: Springer, Cham.

[ref32] Livak K. J. Schmittgen T. D. (2001). Analysis of relative gene expression data using real-time quantitative PCR and the 2−ΔΔCT method. Methods 25, 402–408. doi: 10.1006/meth.2001.1262 11846609

[ref33] Magwanga R. O. Lu P. Kirungu J. N. Cai X. Zhou Z. Wang X. (2018a). Whole genome analysis of cyclin dependent kinase (CDK) gene family in cotton and functional evaluation of the role of CDKF4 gene in drought and salt stress tolerance in plants. Int. J. Mol. Sci. 19. doi: 10.3390/ijms19092625, PMID: 30189594 PMC6164816

[ref34] Meloni D. A. Oliva M. A. Ruiz H. A. Martinez C. A. (2001). Contribution of proline and inorganic solutes to osmotic adjustment in cotton under salt stress. J. Plant Nutr. 24, 599–612. doi: 10.1081/PLN-100104983

[ref35] Michaux C. Martini C. Shioya K. Lecheheb S. A. Budin-Verneuil A. Cosette P.. (2012). CspR, a cold shock RNA-binding protein involved in the long-term survival and the virulence of enterococcus faecalis. J. Bacteriol. 194, 6900–6908. doi: 10.1128/JB.01673-12, PMID: 23086208 PMC3510560

[ref36] Mingkun Y. (2011). Functional Identification and Heterologous Expression of cold shock Proteins in *Deinococcus gobiensis* I-0. dissertation/master's thesis. China (IL): Chinese Academy of Agricultural Sciences.

[ref37] Naidoo G. Naidoo Y. (2001). Effects of salinity and nitrogen on growth, ion relations and proline accumulation in Triglochin bulbosa. Wetl. Ecol. Manag. 9, 491–497. doi: 10.1023/A:1012284712636

[ref38] Nayyar H. Kaur S. Singh S. Upadhyaya H. D. (2006). Differential sensitivity of Desi (small-seeded) and Kabuli (large-seeded) chickpea genotypes to water stress during seed filling: effects on accumulation of seed reserves and yield. J. Sci. Food Agric. 86, 2076–2082. doi: 10.1002/jsfa.2574

[ref39] Omidbakhshfard M. A. Proost S. Fujikura U. Mueller-Roeber B. (2015). Growth-regulating factors (GRFs): a small transcription factor family with important functions in plant biology. Mol. Plant 8, 998–1010. doi: 10.1016/j.molp.2015.01.013, PMID: 25620770

[ref40] Pan J. Wang J. Zhou Z. Yan Y. Zhang W. Lu W.. (2009). IrrE, a global regulator of extreme radiation resistance in Deinococcus radiodurans, enhances salt tolerance in *Escherichia coli* and Brassica napus. PLoS One 4:e4422. doi: 10.1371/journal.pone.0004422, PMID: 19204796 PMC2635966

[ref41] Pingli X. U. Chen L. Zhou X. Daoyi H. E. (2013). The study of Tobacoo transformation with cspB gene from *Bacillus subtilis*. Acta Laser Biology Sinica. 22, 343–353. doi: 10.3969/j.issn.1007-7146.2013.04.010

[ref42] Piya S. Liu J. Burch-Smith T. Baum T. J. Hewezi T. (2020). A role for Arabidopsis growth-regulating factors 1 and 3 in growth-stress antagonism. J. Exp. Bot. 71, 1402–1417. doi: 10.1093/jxb/erz502, PMID: 31701146 PMC7031083

[ref43] Pukale D. D. Farrag M. Gudneppanavar R. Baumann H. J. Konopka M. Shriver L. P.. (2021). Osmoregulatory role of Betaine and Betaine/γ-Aminobutyric acid transporter 1 in post-traumatic Syringomyelia. ACS Chem. Nerosci. 12, 3567–3578. doi: 10.1021/acschemneuro.1c00056, PMID: 34550670

[ref44] Quan R. D. Shang M. Zhang H. Zhao Z. J. (2004). Engineering of enhanced glycine betaine synthesis improves drought tolerance in maize. Plant Biotechnol. J. 2, 477–486. doi: 10.1111/j.1467-7652.2004.00093.x, PMID: 17147620

[ref45] Rontein D. Basset G. Hanson A. D. (2002). Metabolic engineering of osmoprotectant accumulation in plants. Metab. Eng. 4, 49–56. doi: 10.1006/mben.2001.0208, PMID: 11800574

[ref46] Sadau S. B. Ahmad A. Tajo S. M. Ibrahim S. Kazeem B. B. Wei H.. (2021). Overexpression of GhMPK3 from cotton enhances cold, drought, and salt stress in Arabidopsis. Agronomy 11:1049. doi: 10.3390/agronomy11061049

[ref47] Schmid B. Klumpp J. Raimann E. Loessner M. J. Stephan R. Tasara T. (2009). Role of cold shock proteins in growth of listeria monocytogenes under cold and osmotic stress conditions. Appl. Environ. Microbiol. 75, 1621–1627. doi: 10.1128/AEM.02154-08, PMID: 19151183 PMC2655451

[ref48] Shaban M. Miao Y. Ullah A. Khan A. Q. Menghwar H. Khan A. H.. (2018). Physiological and molecular mechanism of defense in cotton against Verticillium dahliae. Plant Physiol. Biochem. 125, 193–204. doi: 10.1016/j.plaphy.2018.02.011, PMID: 29462745

[ref49] Weinberg M. V. Schut G. J. Brehm S. Datta S. Adams M. W. W. (2004). Cold shock of a Hyperthermophilic Archaeon: Pyrococcus furiosus exhibits multiple responses to a suboptimal growth temperature with a key role for membrane-bound glycoproteins. J. Bacteriol. 187, 336–348. doi: 10.1128/jb.187.1.336-348.2005, PMID: 15601718 PMC538827

[ref50] Wyrzykowska J. Pien S. Shen W. H. Fleming A. J. (2002). Manipulation of leaf shape by modulation of cell division. Development 129, 957–964. doi: 10.1242/dev.129.4.957, PMID: 11861478

[ref51] Xia W. Liu X. Xin H. Wu X. Liu R. Li J.. (2021). Saussurea involucrata PIP2;7 Improves Photosynthesis and Drought Resistance by Decreasing the Stomatal Density and increasing intracellular osmotic pressure. Environmental and Experimental Botany.

[ref52] Yamanaka K. Fang L. Inouye M. (1998). The CspA family in *Escherichia coli*: multiple gene duplication for stress adaptation. Mol. Microbiol. 27, 247–255. doi: 10.1046/j.1365-2958.1998.00683.x, PMID: 9484881

[ref53] Yamanaka K. Inouye M. (1997). Growth-phase-dependent expression of CspD, encoding a member of the CspA family in *Escherichia coli*. J. Bacteriol. 179, 5126–5130. doi: 10.1128/jb.179.16.5126-5130.1997, PMID: 9260955 PMC179371

[ref54] Yamanaka K. Mitani T. Ogura T. Niki H. Hiraga S. (1994). Cloning, sequencing, and characterization of multicopy suppressors of a mukB mutation in *Escherichia coli*. Mol. Microbiol. 13, 301–312. doi: 10.1111/j.1365-2958.1994.tb00424.x, PMID: 7984109

[ref55] Yang H. Q. Gao H. J. (2007). Physiological function of arginine and its metabolites in plants, Zhiwu Shengli yu Fenzi Shengwuxue Xuebao. J. Physiol. Mol. Biol. Plants 33, 1–8. doi: 10.1360/aps07042 17287563

[ref56] Yu T. F. Xu Z. S. Guo J. K. Wang Y. X. Abernathy B. Fu J. D.. (2017). Improved drought tolerance in wheat plants overexpressing a synthetic bacterial cold shock protein gene SeCspA. Sci. Rep. 7. doi: 10.1038/srep44050, PMID: 28281578 PMC5345034

[ref57] Yuan M. Chen M. Zhang W. Lu W. Wang J. Yang M.. (2012). Genome sequence and Transcriptome analysis of the Radioresistant bacterium *Deinococcus gobiensis*: insights into the extreme environmental adaptations. PLoS One 7:e34458. doi: 10.1371/journal.pone.0034458, PMID: 22470573 PMC3314630

[ref58] Yuan M. Wei Z. Dai S. Jing W. Wang Y. Tao T.. (2009). *Deinococcus gobiensis* sp nov., an extremely radiation-resistant bacterium. Int. J. Syst. Evol. Microbiol. 59, 1513–1517. doi: 10.1099/ijs.0.004523-0, PMID: 19502345

[ref59] Zhang H. Dong H. Li W. Sun Y. Chen S. Kong X. (2009). Increased glycine betaine synthesis and salinity tolerance in AhCMO transgenic cotton lines. Mol. Breed. 23, 289–298. doi: 10.1007/s11032-008-9233-z

[ref60] Zhang J. H. Huang W. D. Liu Y. P. Pan Q. H. (2005). Effects of temperature acclimation pretreatment on the ultrastructure of mesophyll cells in young grape plants (*Vitis vinifera* L. cv. Jingxiu) Under cross-temperature stresses. J. Integr. Plant Biol. 47, 959–970. doi: 10.1111/j.1744-7909.2005.00109.x

[ref01] Zhang H. Mao L. Xin M. Xing H. Zhang Y. Wu J.. (2022). Overexpression of GhABF3 increases cotton (*Gossypium hirsutum L.*) tolerance to salt and drought. BMC Plant Biol. 22:313. doi: 10.1186/s12870-022-03705-7 35768771 PMC9241229

[ref61] Zhang D. Y. Yang H. L. Li X. S. Li H. Y. Wang Y. C. (2014). Overexpression of Tamarix albiflonum TaMnSOD increases drought tolerance in transgenic cotton. Mol. Breed. 34, 1–11. doi: 10.1007/s11032-014-0015-5

[ref62] Zhang L. Zhang G. Wang Y. Zhou Z. Meng Y. Chen B. (2013). Effect of soil salinity on physiological characteristics of functional leaves of cotton plants. J. Plant Res. 126, 293–304. doi: 10.1007/s10265-012-0533-3, PMID: 23114969

[ref63] Zhu J. K. (2001). Plant salt tolerance. Trends Plant Sci. 6, 66–71. doi: 10.1016/S1360-1385(00)01838-0 11173290

