# Supplementary material for: DgCspC gene overexpression improves cotton yield and tolerance to drought and salt stress comparison with wild-type plants
Source: Front Plant Sci. 2022 Sep 6;13:985900. doi: 10.3389/fpls.2022.985900 (PMC9485673; doi:10.3389/fpls.2022.985900)
Supplement: Supplementary file 1 [file Table_1.DOCX]

Table S1 PCR primers

| Gene name | Primer (5' - 3') |
| --- | --- |
| DgCspC | Forward TGCATGGCTAACGGTAAAGTGAAG |
|  | Reverse TGCTTACCAGCGGTCGTCGCGGCG |

Table S2 Real-time quantitative forward and reverse primers

| Gene name | Primer (5' - 3') |
| --- | --- |
| DgCspC | Forward GGAGAAGGGTTTCGGTTT |
|  | Reverse TTGGTCACGACGATGTTC |
| UBQ7 | Forward GAAGGCATTCCACCTGACCAAC |
|  | Reverse CTTGACCTTCTTCTTCTTGTGCTTG |
| Gh_D08G068000.1  */*HMT-2 | Forward ATGGGTGGAAAATACAGGAGTC |
|  | Reverse GATCTATCGGAAAGTGCCCTG |
| Gh_A05G415500.1  /At4g29890 | Forward AACAGATACAAGAACCCCGTG |
|  | Reverse TCATCCCTGCATACCACAAAC |
| Gh_A11G044900.1  /ALDH10A8 | Forward GGTCAATTGTTCACAGCCATG |
|  | Reverse TTTCAAGTCCCCATTCCCC |
| Gh_D11G045100.1  /ALDH10A8 | Forward TCTGCCAAGCTCCATTCG |
|  | Reverse ACCCCATTGTTCATCTGAGAC |
| Gh_A07G068600.1  /ALDH10A8 | Forward ACTCCCAACCATCAATCCTG |
|  | Reverse GCCCAATCCTTACCTTTGTTTC |
| Gh_A08G073000.1 /HMT-2 | Forward ATGGGTGGAAAATACAGGAGTC |
|  | Reverse GATCTATCGGAAAGTGCCCTG |
| Gh_D04G006200.1  /At4g29890 | Forward AACAGATACAAGAACCCCGTG |
|  | Reverse TTCCCACTTCCAGACACAAG |
| Gh_A02G039800.1  /HMT3 | Forward ATTTGGCTGATGGATCCGAG |
|  | Reverse TCTAGCTTGTTTGGAATTGTTTCG |
| Gh_D02G045900.1 /HMT3 | Forward AACTATGGTGCTTCGGTGAC |
|  | Reverse CCTTGAGAAGCTCTACGTATGC |
| Gh_A08G188100.1  /At4g29890 | Forward ACCTGTGTTTCAGTTCTCGTC |
|  | Reverse TTTCCGCTGTTCTTAGATCCC |
| Gh_D11G286600.1 /Gh_D11G286600 | Forward AGCTGATACGGTTTTGAAGGG |
|  | Reverse ACGGGATGAAGTATAAGCACC |
| Gh_A11G286400.1 /Gh_A11G286400 | Forward TCAAATCCAACCCTAACCCTG |
|  | Reverse CTCGGGTTTGCATGTGTTTG |
| Gh_D11G286700.1 /ODC | Forward TCAAATCCAACCCTAACCCC |
|  | Reverse CTCGGGTTTGCATGTGTTTG |
| Gh_D07G063100.1 /AIH | Forward TTTGCTAGGGTTGCGACTAC |
|  | Reverse TGACATTTGGCGGTAACAGAG |
| Gh_A07G245500.1 /ALDH2B4 | Forward ACTCAGAATATCGAAACCGCC |
|  | Reverse CATCTTGAACCCGCCAAATG |
| Gh_D07G028100.1 /Gh_D07G028100 | Forward ACTGGTGTTGATCCGACTTG |
|  | Reverse GATTTCCACTTACCAGACCCTG |
| Gh_A03G228600.1 /Gh_A03G228600 | Forward AAGGGAAGTTCGGGTTAACG |
|  | Reverse GCGGTTGAAGGGATTTGTG |
| Gh_D02G245200.1 /Gh_D02G245200 | Forward GTCATTTCGGGTCTACTTCGG |
|  | Reverse CGGTTGAAGGGATTTGTGATC |
| Gh_A07G027000.1 /ASP3 | Forward AGTAGCATTTTCTCCCACCTC |
|  | Reverse AGCACCGACTCCTAAATTCAAC |
| Gh_A13G098000.1 /Gh_A13G098000 | Forward TTTCCCATAGTCCCCATTCATC |
|  | Reverse TCAATCTTCCCATCACTGTCAC |
| Gh_A07G195200.1 /POX2 | Forward GACAAGGGTTTCAGAGGGATG |
|  | Reverse GCCAATTCAACACTCCCAAG |
| Gh_D06G049900.1 /ALDH3H1 | Forward ACACGAATGGAAAGCTCAGG |
|  | Reverse CCCATCAAAGCACGCATTATG |
| Gh_A07G062700.1 /AIH | Forward GCGGCTACAATCTCAAAGTTC |
|  | Reverse TGACATTTGGCGGTAACAGAG |
| Gh_A12G275700.2 /SPMS | Forward GGATGGGAAGGTGAATAGTGG |
|  | Reverse GAAGACTGAGATTCCGAGAACC |
| Gh_D13G104800.1 /Gh_D13G104800 | Forward TTTCCCATAGTCCCCATTCATC |
|  | Reverse TCAATCTTCCCATCACTGTCAC |
| Gh_D07G028200.1 /ASP3 | Forward TTTTGATGCTTTGCGTGCTAG |
|  | Reverse CCTCCCATCAGAAGTCATGTAG |
| Gh_A05G023200.1 /PAO5 | Forward TGGTGCTTACTGGGATTCTTG |
|  | Reverse GTTCTCTGGGTGTTTTCATGC |
| Gh_D01G183300.1  /ARB_02965 | Forward TGGCGAAGGATCTGTTTACG |
|  | Reverse TGTTTCGTAGTCTATGCTGGTG |
| Gh_A12G299800.1 /ALDH2B4 | Forward AGAACTTGGAGGGAAATCGC |
|  | Reverse GTGAACAAAAGTGCGAGACC |
| Gh_D05G071600.1 /ALDH3F1 | Forward GCAATCATTCAGTTCGCAGC |
|  | Reverse CCCATGGTATTTCCTATCCCAC |
| Gh_D05G031600.1  /PAO5 | Forward TGGTGCTTACTGGGATTCTTG |
|  | Reverse GTTCTCTGGGTGTTTTCATGC |
| Gh_D12G293500.1 /ALDH2B4 | Forward TTTCAGGAAGGGTGTCGAAC |
|  | Reverse TCCTTTCGTTCCAAGTCTGTC |
| Gh_D09G248800.1 /P4H4 | Forward CTTCAGTCTCCACCCTAATGC |
|  | Reverse TGACCATTTCTCACCTTCGG |
| Gh_A08G165800.1  /PAO1 | Forward AACACCGAAGACACCAATAGAG |
|  | Reverse AACCTCTTTCATCTGCCACC |
| Gh_D07G193100.1 /POX2 | Forward GACAAGGGTTTCAGAGGGATG |
|  | Reverse GCCAATTCAACACTCCCAAG |
| Gh_D06G014100.1 /P4H7 | Forward GAACAGCTGCCAGTAAGTCTAG |
|  | Reverse TGTCTTCAGATCCCTTGCTTC |
| Gh_D06G022400.1  /CYCA3-1 | Forward GTAAAGGGAAGGTGAAAACAAGG |
|  | Reverse TGTGGGTCATTAGACATAGCATC |
| Gh_D05G255500.1  /CYCB1-1 | Forward CTGCTCAGTGTAGTCTTAGTGG |
|  | Reverse CTGTAAGTTTCCCTGTCCCTG |
| Gh_D12G061800.1  /AN3 | Forward GAAATGACCCGTTTTGCCAG |
|  | Reverse TCACAATCTCCCGCCAATC |
| Gh_A09G186000.1  /EBP1 | Forward CCTTCCCCACTTGTCTTTCTG |
|  | Reverse CGCAACTACAGCAATAAACCC |
| Gh_D13G220900.1  /GRF5 | Forward CCTTCCCCACTTGTCTTTCTG |
|  | Reverse CGCAACTACAGCAATAAACCC |
| Gh_D11G228700.1  /EOD | Forward TGGGAATGAAAGGGCTGTATG |
|  | Reverse AGCACGTTTCCTGTTGAGAG |
